# Identifying key items to be addressed by non-clinical operators to manage out-of-hours telephone triage services for older adults seeking non-urgent unplanned care in Belgium: an e-Delphi study

**DOI:** 10.1186/s12913-024-10657-1

**Published:** 2024-02-10

**Authors:** Farah Islam, Pieter Heeren, Kelu Yang, Koen Milisen, Marc Sabbe

**Affiliations:** 1 Department of Public Health and Primary Care, KU Leuven Kapucijnenvoer 35, 3000 Leuven, Belgium; 2 Department of Geriatric Medicine, University Hospitals Leuven, Herestraat 49, 3000 Leuven, Belgium; 3 Department of Emergency Medicine, University Hospitals Leuven, Herestraat 49, 3000 Leuven, Belgium

**Keywords:** Older adults, Delphi study, Non-urgent unplanned care, Out-of-hours telephone triage

## Abstract

**Background:**

This study aimed to achieve expert consensus regarding key items to be addressed by non-clinical operators using computer-software integrated medical dispatch protocols to manage out-of-hours telephone triage (OOH-TT) services for calls involving older adults seeking non-urgent unplanned care across Belgium.

**Methods:**

A three-part classic e-Delphi study was conducted. A purposive sample of experts specialized in out-of-hours unplanned care and/or older persons across Belgium were recruited as panelists. Eligibility criteria included experts with at least 2 years of relevant experience. Level of consensus was defined to be reached when at least 70% of the panelists agreed or disagreed regarding the value of each item proposed within a survey for the top 10 most frequently used protocols for triaging older adults. Responses were analyzed over several rounds until expert consensus was found. Descriptive and thematic analyses were used to aggregate responses.

**Results:**

*N* = 12 panelists agreed that several important missing protocol topics were not covered by the existing OOH-TT service. They also agreed about the nature of use (for the top 10 most frequently used protocols) but justified that some modifications should be made to keywords, interrogation questions, degree of urgency and/or flowcharts used for the algorithms to help operators gain better comprehensive understanding patient profiles, medical habits and history, level of support from informal caregivers, known comorbidities and frailty status. Furthermore, panelists also stressed the importance of considering feasibility in implementing protocols within the real-world setting and prioritizing the right type of training for operators which can facilitate the delivery of high-quality triage. Overall, consensus was found for nine of the top 10 most frequently used protocols for triaging older adults with no consensus found for the protocol on triaging patients unwell for no apparent reason.

**Conclusion:**

Our findings show that overall, a combination of patient related factors must be addressed to provide high quality triage for adults seeking non-urgent unplanned care over the telephone (in addition to age). However, further elements such as appropriate operator training and feasibility of implementing more population-specific protocols must also be considered. This study presents a useful step towards identifying key items which must be targeted within the larger scope of providing non-urgent out-of-hours telephone triage services for older adults seeking non-urgent unplanned care.

**Supplementary Information:**

The online version contains supplementary material available at 10.1186/s12913-024-10657-1.

## Introduction

It is well established that the use of out-of-hours telephone triage services (OOH-TT) services has large potential for managing unplanned and OOH healthcare needs presented by the general population [1, 2]. However, little is known with regards to the quality of these services in more vulnerable segments of the population such as older adults [1, 3, 4]. Specifically, it is well known that older adults (over the age of 65) are especially difficult to triage over TT services as they frequently present with atypical symptoms of common health concerns. At the same time, these segments of the population not only make up a large proportion of all unplanned care users [5, 6] but they are also known to be at the highest risks for experiencing adverse health events and safety incidents due to inappropriate care transfers [7] and in cases of inappropriate triage, mis-triage [8, 9].

As a first step towards addressing this issue, a recent publication by Wardlow et al., (2022) aimed to better understand how telehealth-based services should be delivered to appropriately meet the needs of older adults seeking care [10]. However, existing findings have largely focused on provider-focused principles and guidelines for promoting age-friendly telehealth practices [10]. Furthermore, some additional recent efforts also found that developing or adapting existing protocols to better fit population-specific criteria for triage in older adults may be effective for reducing rates of mis-triage in older adults. To date, these efforts have only been examined in specific and in-person triage tools [11–13] and it is not yet clear how well they may apply within the larger context of medical dispatch protocols used by OOH-TT services.

In Belgium, there is a national OOH-TT service which aims to provide medical assistance to patients during evenings, weekends and holidays. Depending on the region, patients calling this number are re-directed to the local on-call general practitioner (GP) for an appointment or to a non-medical operator specialized in triage using the Belgian Manual for Medical Regulation (BHMR) for guidance on further medical assistance (more information available at: www.1733.be (Dutch, French, German and English) [3, 14, 15]. Overall, the use of these medical dispatch protocols have been shown to be safe and effective for use in the general population [16–18]. However, lower quality of triage (particularly related to concerns of safety assessed via the frequency of adverse events, errors, and patient hospitalization rates) has been shown to be associated with triaging older adults over the telephone [19–21]. One potential problem associated with the use of existing protocols, such as the BHMR is the generic nature of the protocols. It is typically the case that the same protocols are used for triaging all segments of the population (i.e. including children and older adults who are known to be at higher risks for lower quality triage) with some limited considerations of age and medical history for vulnerable segments of the population. However, by incorporating key population-specific items within the protocols, the potential effectiveness, efficiency and overall quality of managing calls involving older adults in the population may be improved. As such, this Delphi study aims to achieve expert consensus regarding key items to be addressed by non-clinical operators using the BHMR medical dispatch protocols to manage OOH-TT services for calls involving older adults seeking non-urgent unplanned care across Belgium.

## Methods

### Study design

A three-part classic e-Delphi study was conducted to address the aim of our research through an iterative process which seeks to combine diverse expert opinions into group consensus based on the guidance of Keeney et al. (2011) and recommendations for Delphi study reporting guidelines in the health science sector by Spranger et al. (2022) [22–24].

### Expert panel recruitment and selection

A purposive sample of *N* = 16 experts specialized in out-of-hours unplanned care and/or older persons across Belgium were recruited for participation as panelists in our study via the network of the research team. Identified experts were first contacted via the telephone and were given some brief information regarding the study [22]. Panelists who agreed to participate were then contacted electronically (via e-mail) with a formal invitation letter and a complete study information sheet. Panelists were also informed that their participation is voluntary and that they were free to withdraw from the study at any time.

### Eligibility criteria

Eligibility criteria for panelists included experts with at least 2 years of prior professional experience relevant to unplanned care for older adults and/or telephone triage including emergency physicians, nurses, GPs, geriatricians, telephone triage operators, patient representatives and medical directors of OOH-TT services across Belgium.

### BHMR protocols

All calls received by the 1733 service are managed by non-clinical operators who are trained to use the BHMR protocols; a set of computer-software integrated medical protocols (more info: https://www.health.belgium.be/nl/belgische-handleiding-voor-de-medische-regulatie#anchor-25165) [15, 25]. The use of the BHMR protocols by 1733 service operators is mandatory. The protocols examined in this study includes version 4.0 (published on 5 June, 2019) which consists of 56 different protocols that can be prompted by entering specific key words and descriptions of medical symptoms [25]. Although these protocols are largely generic, some considerations are made for specific cases involving children between the ages of 0–14, adults aged 75 and over and patients with chronic illnesses.

### Data collection procedures

This Delphi study consisted of three parts. The first two parts of the study were held electronically and the third part was held via a face-to-face consensus meeting with panelists. All surveys were pilot tested before administered to panelists. During part one and part two, panelists were asked to fill out online surveys using Qualtrics software.

###  Part one (survey one)

Following consent to participate, participants were sent an e-mail link to complete a first online survey. The first part aimed to collect demographic information of panelists. The remainder of the survey was composed of open- and closed- ended information regarding panelists beliefs surrounding current protocols used by the 1733 service for triaging older adults seeking OOH unplanned non-urgent care. Specifically, panelists were presented with the list of protocols that are available at the 1733 service and were asked whether they believe the protocol topics considered in the existing list to be sufficient for triaging older adults as well as to self-report (based on their expert experiences) on any relevant protocol topic they believed to be missing from the existing list. Panelists were then presented with the 10 most frequently used protocols for triaging older adults via the 1733 OOH-TT service between January 1 to December 31, 2019. Complete algorithms, flowcharts, and frequency distributions were made available to enable a detailed analysis of each of the individual protocols (see Table 1) [25].

**Table 1:** Summary of protocol topics available for use within the 1733 OOH-TT service

#	Topic title
**1.** 2. 3. 4. 5. 6. 7. **8.** 9. 10. 11. 12. 13. 14. 15. **16.** 17. 18. 19. 20. 21. 22. 23. 24. 25. **26.** **27.** **28.** **29.** 30. 31. **32.** 33. 34. 35. 36. 37. 38. 39. 40. 41. 42. 43. **44.** **45.** 46. 47. 48. 49. 50. 51. 52. 53. 54. 55. 56.	**Breathing difficulties (1)** Aggression - fight - rape Allergic reaction Unconscious - coma - syncope Bite wounds Bleeding/blood loss Burns **Cardiac problem other than thoracic pain (7)** Carbon monoxide poisoning Communication with on-call medical doctor Control police – alcohol/drugs Transient ischemic attack - Stroke Dizziness Electrocution Epilepsy - convulsions **Cardiac arrest – deceased (5)** Heat stroke - sunstroke Skin problems Headache Alcohol poisoning Poisoning with recreational drugs Poisoning with household, agricultural or industrial products Poisoning with medication Febrile seizures child < 7 years Severe crush injury **Hot or cold limb (9)** **Nose-throat-ear-tooth problem (8)** **Non-traumatic abdominal pain (2)** **Non-traumatic back pain (10)** Airway Obstruction Oncology patient under treatment **Unwell for no apparent reason (3)** Eye problems Palliative patient Patient with defibrillator or pacemaker Patient does not answer the call Chest pain Sudden deafness and ringing in the ears Postop problems Psychiatric problems Skull trauma Social problems Diabetes - diabetes **Trauma (4)** **Urogenital problems (6)** Fall from a great height (> 3 m) Drowning - diving accident Hanging and/or strangulation Traffic accident Wound by weapon Wound Sick baby < 3 months (infant) Sick child < 15 years with abdominal pain 5Sick child < 15 years with fever Sick child < 15 years with respiratory infection Pregnancy - childbirth

Text in bold indicates top 10 most frequently used protocols used for triaging older adults via the 177 service between January 1 2019 to December 31, 2019

After review of each protocol, panelists were asked to provide their opinion regarding the frequency of use and content of protocols for triaging calls involving older adults seeking unplanned care. Panelists were asked whether each protocol was used as frequently as they would expect and whether they believed that any modifications should be made to the content (i.e. the keywords, interrogation questions, degree of urgency, flowchart, or other factors related to the algorithm) within a specific protocol to improve the management of calls involving older adults.

###  Part two (survey 2)

Panelists who completed part one of the survey were invited to partake in the second survey. The results of the first survey were used to form the basis of the second survey. Panelists were presented with a summary of (de-identified) responses that were submitted. Using a four-point Likert scale, they were asked to rate each of the submitted responses (considered as *items*) as “not relevant at all”; “somewhat relevant”; “quite relevant”; or “extremely relevant.” An open comments box was provided for panelists to further specify each of their responses (i.e. to elaborate on responses, or to add further suggestions and/or comments). Once all the responses were submitted, ratings for each item were analyzed for consensus and were re-submitted to panelists as necessary.

###  Part three (consensus meeting)

The consensus meeting aimed to achieve final consensus on items identified for each topic rated during part one and part two. The consensus meeting was held using a hybrid format and all panelists were invited to join the meeting in person or online based on their availability.

### Predefined criteria for consensus

In line with expert recommendations, level of consensus was defined a priori to be reached when at least 70% of the expert panel members agreed (positive consensus defined as the item being generally scored as “quite relevant” or “extremely relevant”) or disagreed (negative consensus defined as the item being generally scored as “not relevant at all” or “somewhat relevant”) regarding the relevance of each item proposed by panelists within a survey [22]. If consensus was not reached following first-time analysis of responses obtained for an item, the survey was re-administered for a second round and panelists were requested to re-submit their responses based on group results obtained during the previous round. If consensus was not reached by the second round, then the item was to be further discussed for a final time during the round table consensus meeting.

### Data analyses

Responses were quantitively and qualitatively analyzed over several rounds until expert consensus was found. Descriptive and thematic analyses were used to aggregate responses. Qualtrics was used for data gathering and analysis while SPSS Statistics (version 24.0.0.0) was used for analysis purposes.

### Ethical considerations

This project received ethical approval from the Medical Ethics Committee UZ KU Leuven (study reference number S64473).

## Results

**Fig. 1:**
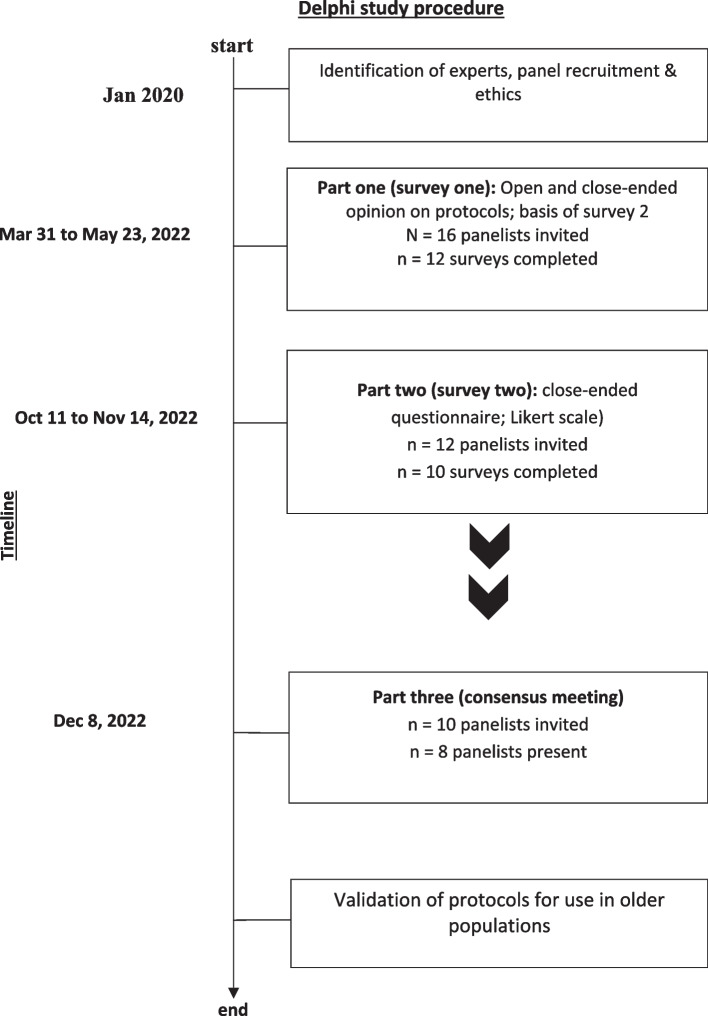
Timeline of Delphi study procedure

Data was collected between March 31, 2022 until December 8, 2022. A total of *N* = 16 panelists were invited to complete survey one, out of which 12 responses were obtained followed by 10 responses for survey two. Eight panelists were present for the consensus meeting (see Fig. 1). The expert panel was comprised of *n* = 1 medical director of a call center, *n* = 2 GPs, *n* = 2 emergency physicians, *n* = 2 geriatricians, *n* = 3 telephone triage operators, *n* = 2 nurses, *n* = 1 other (telephone triage center coordinator) (panelists could select more than one category for multiple professional backgrounds and/or area of work). The majority of panelists worked in the field of academia (*n* = 6), or in a clinical setting (*n* = 7) and half (*n* = 6) had between 11 to 30 years of work experience. (see Table 2).

**Table 2:** Expert panel characteristics

Characteristic	n	(%)
Professional background		
Medical director of a call center	1	(7.7)
General practitioner	2	(15.4)
Emergency department physician	2	(15.4)
Geriatrician	2	(15.4)
Telephone triage operator	3	(23)
Nurse	2	(15.4)
Patient representative	0	(0)
Other	1	(7.7)
Total	13	(100)
Area of work		
Academia/research	6	(31.6)
Clinical	7	(36.8)
Government	3	(15.8)
Call center	3	(15.8)
Other	0	(0)
Total	19	(100)
Years of experience		
0–10	5	(41.7)
11–20	3	(25.0)
21–30	3	(25.0)
31–40	1	(8.3)
Total	12	(100)
Region of work in Belgium		
Flanders	9	(75.0)
Wallonia	2	(16.7)
Brussels	1	(8.3)
Total	12	(100)

Note: Panelists could select more than one category for the following variables: professional background, area of work

### Survey 1

In total, *n* = 8 panelists responded that they believe there to be several self-reported missing protocol topics which are not currently covered by the existing BHMR protocols but that should be added to improve the quality of triage for calls involving older adults ≥65 years of age seeking unplanned care. The following categories of missing protocol topics were reported to include: *Skull Trauma, Generalized Fever In Adults, Medication, Neurological Problems Other than stroke, fever without an infection focus, Fall-Related, Neglect, Acute Confusion, Non-Traumatic Joint Pains, Reduced Functionality and Decline of General Conditions.* Overall, at least half or more panelists also reported that all of the top 10 most frequently used protocols for triaging older adults via the 1733 OOH-TT service were used as frequently as they would expect (see Table 3)**.** At the same time, at least half or more panelists felt that some modifications should be made to the content of all the 10 protocols to improve the overall management of calls involving older adults (see Table 4 and Table 5).

**Table 3:** Summary of 10 most frequently used protocol topics presented to panelists and frequency of use

#	Protocol title	Frequency of use									
		Used as expected		Under-used		Over-used		Not sure		Total	
		n	(%)	n	(%)	n	(%)	n	(%)	N	(%)
1.	Breathing Difficulties	10	(90.9)	1	(9.1)	0	(0)	0	(0)	11	(100)
2.	Non-Traumatic Abdominal Pain	9	(81.8)	1	(9.1)	1	(9.1)	0	(0)	11	(100)
3.	Unwell for no apparent reason	2	(18.2)	4	(36.4)	4	(36.4)	1	(9.1)	11	(100)
4.	Trauma	8	(72.7)	2	(18.2)	1	(9.1)	0	(0)	11	(100)
5.	Cardiac arrest – deceased	7	(63.6)	2	(18.2)	0	(0)	2	(18.2)	11	(100)
6.	Urogenital problems	9	(81.8)	1	(9.1)	0	(0)	1	(9.1)	11	(100)
7.	Cardiac problem other than thoracic pain	6	(54.5)	2	(18.2)	0	(0)	3	(27.3)	11	(100)
8.	Nose-throat-ear-tooth	6	(54.5)	1	(9.1)	2	(18.2)	2	(18.2)	11	(100)
9.	Hot or cold limb	6	(54.5)	2	(18.2)	2	(18.2)	1	(9.1)	11	(100)
10.	Non-traumatic back pain	7	(63.6)	2	(18.2)	1	(9.1)	1	(9.1)	11	(100)

Note: One respondent did not provide any answers to this question (*N* = 12)

**Table 4:** Summary of panelist responses on whether content related modifications to be made for each protocol

#	Protocol title	Support for content-related modifications					
		Yes		No			Total
		n	(%)	n	(%)	N	(%)
1.	Breathing Difficulties	6	(60)	4	(40)	10	(100)
2.	Non-Traumatic Abdominal Pain	7	(70)	3	(30)	10	(100)
3.	Unwell for no apparent reason	5	(50)	5	(50)	10	(100)
4.	Trauma	7	(70)	3	(30)	10	(100)
5.	Cardiac arrest – deceased	6	(60)	4	(40)	10	(100)
6.	Urogenital problems	6	(60)	4	(40)	10	(100)
7.	Cardiac problem other than thoracic pain	4	(40)	6	(40)	10	(100)
8.	Nose-throat-ear-tooth	5	(50)	5	(50)	10	(100)
9.	Hot or cold limb	5	(50)	5	(50)	10	(100)
10.	Non-traumatic back pain	7	(70)	3	(30)	10	(100)

Note: Two respondents did not provide any answers to this question (*N* = 12)

**Table 5:** Summary of panelist responses supporting content related modifications to be made for each protocol

#	Protocol title	Support for content-related modifications									
		Keywords		Interrogation questions		Degree of urgency		Flowchart		Other	
		n	(%)	n	(%)	n	(%)	n	(%)	n	(%)
1.	Breathing Difficulties	3	(21.4)	4	(28.6)	3	(21.4)	3	(21.4)	1	(7.1)
2.	Non-Traumatic Abdominal Pain	1	(10)	3	(30)	2	(20)	4	(40)	0	(0)
3.	Unwell for no apparent reason	3	(33.3)	2	(22.2)	3	(33.3)	1	(11.1)	0	(0)
4.	Trauma	4	(36.4)	4	(36.4)	0	(0)	2	(18.2)	1	(9.1)
5.	Cardiac arrest – deceased	1	(10)	4	(40)	2	(20)	0	(0)	3	(30)
6.	Urogenital problems	2	(20)	4	(40)	1	(10)	2	(20)	1	(10)
7.	Cardiac problem other than thoracic pain	2	(20)	30	(30)	1	(10)	3	(30)	1	(10)
8.	Nose-throat-ear-tooth	2	(28.6)	2	(28.6)	0	(0)	1	(14.3)	2	(28.6)
9.	Hot or cold limb	1	(20)	2	(40)	1	(20)	1	(20)	0	(0)
10.	Non-traumatic back pain	2	(15.4)	4	(30.8)	4	(30.8)	2	(15.4)	1	(7.7)

Note: Panelists could select more than one category for sections of content related modifications to be made for each protocol

### Survey two and consensus meeting

The top 10 most frequently used protocols for triaging older adults were assessed and discussed by panelists during survey two and the consensus meeting (see Table 6 below as an example with complete details available in Supplementary Tables 1 (survey two results) and 2 (consensus meeting results)). In total, consensus (positive or negative) was found for all items on two of the protocols (namely “Trauma” and “Cardiac arrest- deceased”) during survey two. Consensus was not found for the remaining eight protocols.

**Table 6:** Example of findings for protocol on “Breathing Difficulties”

Survey two results											
	Relevance										Consensus status
Item	Not relevant at all		Somewhat relevant		Quite relevant		Extremely relevant		Total		Yes/no (positive/negative)
	n	(%)	n	(%)	n	(%)	n	(%)	N	(%)	
1. Symptoms specific for older adults/geriatric patients	1	(10)	1	(10)	6	(60)	2	(20)	10	(100)	Yes (positive)
2. Coughing	1	(10)	4	(40)	4	(40)	1	(10)	10	(100)	No
3. Coloured sputa	1	(10)	3	(30)	4	(40)	2	(20)	10	(100)	No
4. Fever	0	(0)	4	(40)	3	(30)	3	(30)	10	(100)	No
5. Symptoms related to heart failure	0	(0)	1	(10)	4	(40)	5	(50)	10	(100)	Yes (positive)
Consensus meeting results											
	Relevance										Consensus status
Item	Not relevant at all		Somewhat relevant		Quite relevant		Extremely relevant		Total		Yes/no (positive/negative)
	n	(%)	n	(%)	n	(%)	n	(%)	N	(%)	
1. Symptoms specific for older adults/geriatric patients	Consensus obtained during survey two										Yes (positive)
2. Coughing	0	(0)	1	(13)	2	(25)	5	(63)	8	(100)	Yes (positive)
3. Coloured sputa	0	(0)	0	(0)	1	(13)	7	(88)	8	(100)	Yes (positive)
4. Fever	Excluded from final round table discussion^a^										N/A
5. Symptoms related to heart failure	Consensus obtained during survey two / Excluded from final round table discussion^a^										Yes (positive

*N/A* not applicable

Note: ^a^suggestions included in new 1733 protocols

The remaining eight protocols were further discussed during the consensus meeting. One protocol (namely, the protocol on “Non-traumatic back pain”) was excluded from discussion because several panelists commented that the items addressed in this protocol were already addressed in the updated version of the BHMR protocols (version 5.0) which was released for use by the 1733 service during the data collection period of this study [26]. Full consensus was found for all items on the remaining protocols except the protocol “Unwell for no apparent reason.” Overall, panelists stressed that a combination of patient related factors must be addressed to provide high quality triage for adults seeking non-urgent unplanned care over the telephone (in addition to age) including patient profiles, medical habits and history, level of support from informal caregivers, known comorbidities and frailty status. Additionally, panelists also highlighted that further elements such as appropriate operator training and feasibility of integrating revised protocols (i.e. more population-specific protocols) must also be considered before implementation of protocols within the call center. The following specific outcomes were obtained for each protocol item (see details in Supplementary Tables 1 and 2):

### Protocol on “Trauma”

A total of four items were addressed for the protocol on “Trauma”. Positive consensus was found for all items including *fall in older adults, location of injury, reason for fall, and level of urgency***.**

### Protocol on “Cardiac arrest- deceased”

A total of two items were addressed for the protocol on “Cardiac arrest – deceased”. Positive consensus was found for all items including *nature of death* and *practical questions to better evaluate dispatch to the Mobile Emergency Group*.

### Protocol on “Breathing Difficulties”

A total of five items were addressed for the protocol on “Breathing Difficulties”. Two items including *fever* and *symptoms related to heart failure* were excluded from the round table discussion as these suggestions were already included in the new version of the BHMR protocols [26]. Positive consensus was found for the remaining items including *symptoms specific for older adults/geriatric patients, coughing* and *coloured sputa***.**

### Protocol on “Non-Traumatic Abdominal Pain”

A total of six items were addressed for the protocol on “Non-Traumatic Abdominal Pain”. Four items including *abnormal aorta, aneurism alertness, diarrhea* and *pain severity* were excluded from the round table discussion as these suggestions were already included in the new version of the BHMR protocols [26]. Positive consensus was found for the item *hydration level* and negative consensus was found for the item *history about previous aneurysm***.**

### Protocol on “Urogenital Problems”

A total of five items were addressed for the protocol on “Urogenital Problems”. Positive consensus was found for the items on p*roblems with use of other medical devices in older adults ≥ 65*, *urinary incontinence, urinary overflow* and *possibility to send patient a GP for home visit.* Negative consensus was found for the item *removal of questions related to temperature and urinary retention from this protocol and added to “confusion” protocol instead.*

### Protocol on “Cardiac Problem Other Than Thoracic Pain”

A total of nine items were addressed for the protocol on “Cardiac Problem Other Than Thoracic Pain”. Two items including *addition of swollen legs complaint to protocol for hot/cold limbs* and *possibility to push dimple into swollen leg (possibly indicating heart failure)* were excluded from the round table discussion as these suggestions were already included in the new version of the BHMR protocols [26]. Of the seven remaining items, positive consensus was found for the items *adapting current 1733 protocol for “syncope” to broader guidelines for “transit loss of consciousness* and *possibility to push dimple into leg given shortness of breath”*. Negative consensus was found for the items on *swollen leg in older adults ≥ 65, shortness of breath in older adults ≥ 65, irregular heart palpitations in older adults ≥ 65, respiratory problems in older adults ≥ 65*, and *orthostatic hypotension.*

### Protocol on “Nose-Throat-Ear-Tooth”

A total of four items were addressed for the protocol “Nose-Throat-Ear-Tooth.” Positive consensus was found for the items *problem with swallowing foods* and *clarification of GP’s role for patients with tooth problem.* Negative consensus was found for the item*s tooth problems for all ages (separate from nose-throat-ear protocol)* and *location of pain.*

### Protocol on “Hot or Cold Limb”

In total, three items were addressed for the protocol “Hot or cold limb.” Two items including *swollen leg joints for all ages* and *integration of swollen legs or joints complaint intro current protocol for hot or cold limb* were excluded from the round table discussion as these suggestions were already included in the new version of the BHMR protocols [26]. Positive consensus was found for the remaining item on *pain severity and changes in pain severity when lifting leg up and down.*

### Protocol on “Unwell for No Apparent Reason”

In total, eight items were addressed for the protocol “Unwell for no apparent reason.” Positive consensus was found for the items on *unwellness (for no apparent reason) in older adults ≥ 65, fever without focus in older adults ≥ 65, acute confusion in older adults ≥ 65, pain and mobility in older adults ≥ 65* and *temperature* and *urinary retention,* and for the item *atypical symptoms in older adults ≥ 65* and *dysregulated blood pressure*. No consensus was found by panelists for the item on *voluntary stopping of eating and drinking*.

## Discussion

This study presents one of the first scientific efforts to better understand and find expert consensus regarding key items to be addressed by non-clinical operators using computer-software integrated medical dispatch protocols to manage OOH-TT services for older adults seeking unplanned care. Our interdisciplinary expert panel found strong consensus for the majority of the 10 most frequently used BHMR protocols for triaging older adults within the larger 1733 service in Belgium. Findings showed that the majority of experts felt that most protocols used for managing calls involving older adults were used as frequently as they would expect. However, panelists also agreed that some content related modifications should be made for keywords, interrogation questions, degree of urgency and/or flowcharts used for the algorithms of the top 10 most frequently used protocols to improve the overall quality of triage for older adults.

Overall implications of our research suggest that in addition to age of patients, a combination of patient related factors must be addressed to provide high quality triage for adults seeking non-urgent unplanned care over the telephone. Additionally, some further elements such as appropriate operator training and feasibility of implementing more population-specific protocols must also be considered within the call center setting. Such considerations must be thoroughly evaluated before implementation of any new or revised protocols within practice.

Specifically, our findings shows that overall, a combination of patient related factors must be addressed to provide high quality triage for adults seeking non-urgent unplanned care over the telephone (in addition to age). However, further elements such as appropriate operator training and feasibility of implementing more population-specific protocols must also be considered. This study presents a useful step towards identifying key items which must be targeted within the larger scope of providing non-urgent out-of-hours telephone triage services for older adults seeking non-urgent unplanned care.

Despite known concerns of triaging older adults via OOH-TT services, currently existing methods are still largely catered for providing remote medical assistance to less vulnerable segments of the population. It is not yet clearly known how protocols used across various services may be adapted and/or optimized for use in older adults [3, 17, 18, 22, 23, 25, 27]. One plausible explanation for this concern as discussed during the round table consensus meeting is the understanding that it is especially difficult (and perhaps not possible) to create age specific protocols for older adults in a way that is similar to children in which specific ages are often lined with specific pathologies [28]. The importance of addressing triage for older adults by using methods in which operators prioritize assessing a combination of factors (including patient profiles, medical habits and history, level of support from informal caregivers, known comorbidities and frailty status) rather than solely evaluating differences in patient age was embedded throughout the discussion.

Another key point that was addressed by panelists is the feasibility of implementing new/revised protocols within real world settings. Panelists were largely opposed to the creation of new protocols for providing OOH-TT services to older adults due to time and clinical restrictions of the telephone operators. While the importance of providing adequate training for operators was largely agreed upon, the key focus mentioned by panelists was to consider the addition and integration of specific questions that may capture different dimensions of triage-related questions for calls involving older adults within currently existing protocols. Despite a lack of existing literature, [29] discussions on evaluating quality of training in OOH-TT operators were also highlighted to be important and in line with the surrounding literature base which has shown that operator related characteristics (such as clinical background, length of experience, type of previous experience and flexibility of decision-making regarding level of urgency) may be key factors which influence triage outcome [3, 4, 30–33]. It is therefore crucial to ensure that operators are given adequate training which can in turn, ensure consistency and high-quality dispatch of patients using OOH-TT services. Supplementing the right questions alongside well-trained operators who are able to gather the right information during a call was stressed by panelists to be a key combination for enabling the delivery of high-quality OOH-TT services for older adults.

Several strengths and limitations must be considered in interpreting the overall study results. First, it is important to note that our conclusions provide insight into discussions and consensus drawn by one particular expert panel and do not necessarily reflect objectively correct answers which must be used for delivering OOH-TT services to older adults. It is also important to note that different reflections and ideas regarding quality of triage may be produced by panelists consisting of other experts [27, 34]. As such, the results of this study must be interpreted with caution. Furthermore, level of consensus in this study was defined to be reached when at least 70% of the expert panel members agreed or disagree regarding the value of a specific item. Although this is within the standard range of measuring consensus [22], it must be noted that an alternative definition of level of consensus may yield different results. A key strength of this Delphi study is that the process inherently allows flexibility and enables panelists to assess their judgements through several rounds of the research process [30]. Furthermore, it also plays a vital role for linking together existing knowledge and areas of agreement and disagreement within this field of research through a structured process that can allow us to ultimately have an impact on improving the quality of OOH-TT protocols for use in older adults [31, 35]. A limitation that must also be considered is that no views of patient representatives were included. Despite several attempts to reach out to various eligible contacts, we were not able to include any panelists who could represent the views of older adults who have experience with the use of OOH-TT services. Finally, it is important to mention that although we intended for a national representative Delphi panel (and has therefore reached out to panelists from three regions in Belgium – Flanders, Wallonia, and Brussels), we were presented with a high dropout rate of French speaking panelists leaving us with a small expert panel of 8 participants for the final consensus round. Furthermore, the study team moderating the development of the questionnaires and consensus meeting were all based in a university and/or clinical settings located in Flanders. As such, the results are therefore biased towards expert views of panelists from the Flemish region (i.e. Flanders) of Belgium. However, given the rather flexible nature of a Delphi study to determine sample size and heterogeneity based on the larger study aim and possible range of opinions, we believe that that concern of mathematical/statistical bias is limited.

## Conclusion

This Delphi study presents one of the first scientific efforts targeting the improvement of TT protocols used for triaging older adults seeking non-urgent out-of-hours unplanned care. Overall, this research is a first step towards addressing current gaps in the existing topics of the BHMR and for identifying important content related benchmarks which must be targeted within the larger scope of OOH-TT services. Our findings showed that overall, a combination of patient related factors must be addressed to provide high quality triage for adults seeking non-urgent unplanned care over the telephone (in addition to age). However, further elements such as appropriate operator training and feasibility of implementing more population-specific protocols must also be considered. This is a useful step towards identifying key items which must be targeted within the larger scope of providing non-urgent out-of-hours telephone triage services for older adults seeking non-urgent unplanned care. We hope that these findings will eventually be used for guiding quality improvements within the BHMR protocols used within the context of the 1733 service but also for similar OOH-TT settings internationally.

### Supplementary Information

**Additional file 1.**

**Additional file 2.**

## Data Availability

For any requests about data generated or analyzed during this study, please contact the corresponding author.

## Abbreviations

OOH: Out-of-hours
TT: Telephone triage
ED: Emergency department
BHMR: Belgian Manual for Medical Regulation
