# Supplementary material for: Identifying key items to be addressed by non-clinical operators to manage out-of-hours telephone triage services for older adults seeking non-urgent unplanned care in Belgium: an e-Delphi study
Source: BMC Health Serv Res. 2024 Feb 10;24:189. doi: 10.1186/s12913-024-10657-1 (PMC10858535; doi:10.1186/s12913-024-10657-1)
Supplement: Supplementary file 1 — Additional file 1. [file 12913_2024_10657_MOESM1_ESM.docx]

| **Supplementary Table 1:** Survey two results  Protocol on “Breathing Difficulties” | | | | | | | | | | | | | | | | |  | |
| --- | --- | --- | --- | --- | --- | --- | --- | --- | --- | --- | --- | --- | --- | --- | --- | --- | --- | --- |
|  | | | | Relevance | | | | | | | | | | | | | Consensus status | |
| Item |  |  |  | Not relevant at all | | Somewhat relevant | | Quite relevant | | | Extremely relevant | | | Total | | | Yes/no (positive/negative) | |
|  |  |  |  | n | (%) | n | (%) | | n | (%) | | n | (%) | | N | (%) | |  |
| 1. Symptoms specific for older adults/geriatric patients | | | | 1 | (10) | 1 | (10) | | 6 | (60) | | 2 | (20) | | 10 | (100) | | Yes (positive) |
| 1. Coughing | | | | 1 | (10) | 4 | (40) | | 4 | (40) | | 1 | (10) | | 10 | (100) | | No |
| 1. Coloured sputa | | | | 1 | (10) | 3 | (30) | | 4 | (40) | | 2 | (20) | | 10 | (100) | | No |
| 1. Fever | | | | 0 | (0) | 4 | (40) | | 3 | (30) | | 3 | (3) | | 10 | (100) | | No |
| 1. Symptoms related to heart failure | | | | 0 | (0) | 1 | (10) | | 4 | (40) | | 5 | (50) | | 10 | (100) | | Yes (positive) |
|  | | | | | | | | | | | | | | | | |  | |

|  | | | | | | | | | | | | | | | | |  | |
| --- | --- | --- | --- | --- | --- | --- | --- | --- | --- | --- | --- | --- | --- | --- | --- | --- | --- | --- |
| Protocol on “Non-Traumatic Abdominal Pain” | | | | | | | | | | | | | | | | |  | |
|  | | | | Relevance | | | | | | | | | | | | | Consensus status | |
| Item |  |  |  | Not relevant at all | | Somewhat relevant | | Quite relevant | | | Extremely relevant | | | Total | | | Yes/no (positive/negative) | |
|  |  |  |  | n | (%) | n | (%) | | n | (%) | | n | (%) | | N | (%) | |  |
| 1. History about previous aneurysm | | | | 0 | (0) | 4 | (40) | | 4 | (40) | | 2 | (20) | | 10 | (100) | | No |
| 1. Abnormal aorta aneurism (need to exclude this) | | | | 0 | (0) | 5 | (55.6) | | 2 | (22.2) | | 2 | (22.2) | | 9 | (100) | | No |
| 1. Alertness | | | | 0 | (0) | 4 | (40) | | 3 | (30) | | 3 | (30) | | 10 | (100) | | No |
| 1. Hydration level | | | | 1 | (10) | 2 | (20) | | 6 | (60) | | 1 | (10) | | 10 | (100) | | Yes (positive) |
| 1. Diarrhea | | | | 0 | (0) | 4 | (40) | | 5 | (50) | | 1 | (10) | | 10 | (100) | | No |
| 1. Pain severity | | | | 0 | (0) | 4 | (40) | | 3 | (30) | | 3 | (30) | | 10 | (100) | | No |
|  | | | | | | | | | | | | | | | | |  | |

|  | | | | | | | | | | | | | | | | |  | |
| --- | --- | --- | --- | --- | --- | --- | --- | --- | --- | --- | --- | --- | --- | --- | --- | --- | --- | --- |
| Protocol on “Unwell for no apparent reason” | | | | | | | | | | | | | | | | |  | |
|  | | | | Relevance | | | | | | | | | | | | | Consensus status | |
| Item |  |  |  | Not relevant at all | | Somewhat relevant | | Quite relevant | | | Extremely relevant | | | Total | | | Yes/no (positive/negative) | |
|  |  |  |  | n | (%) | n | (%) | | n | (%) | | n | (%) | | N | (%) | |  |
| 1. Unwellness (for no apparent reason) in older adults ≥ 65 | | | | 2 | (20) | 3 | (30) | | 3 | (30) | | 2 | (20) | | 10 | (100) | | No |
| 1. Fever without focus in older adults ≥ 65 (i.e high temperature as the only presenting feature) | | | | 0 | (0) | 5 | (50) | | 1 | (10) | | 4 | (40) | | 10 | (100) | | No |
| 1. Acute confusion in older adults ≥ 65 | | | | 0 | (0) | 1 | (10) | | 6 | (60) | | 3 | (30) | | 10 | (100) | | Yes (positive) |
| 1. Pain and mobility in older adults ≥ 65 | | | | 1 | (10) | 3 | (30) | | 4 | (40) | | 2 | (20) | | 10 | (100) | | No |
| 1. Atypical symptoms in older adults ≥ 65 | | | | 2 | (20) | 3 | (30) | | 2 | (20) | | 3 | (30) | | 10 | (100) | | No |
| 1. Fever and length of fever 2. Voluntary stopping of eating and drinking | | | | 1  0 | (10)  (0) | 3  4 | (30)  (40) | | 4  5 | (40)  (50) | | 2  1 | (20)  (10) | | 10  10 | (100)  (100) | | No  No |
| 1. Dysregulated blood pressure | | | | 1 | (1) | 4 | (40) | | 5 | (50) | | 0 | (0) | | 10 | (100) | | No |
| 1. Temperature and urinary retention (related to confusion) | | | | 0 | (0) | 3 | (33.3) | | 1 | (11.1) | | 5 | (55.6) | | 9 | (100) | | No |
|  | | | | | | | | | | | | | | | | |  | |

| Protocol on “Trauma” | | | | | | | | | | | | | | | | |  | |
| --- | --- | --- | --- | --- | --- | --- | --- | --- | --- | --- | --- | --- | --- | --- | --- | --- | --- | --- |
|  | | | | Relevance | | | | | | | | | | | | | Consensus status | |
| Item |  |  |  | Not relevant at all | | Somewhat relevant | | Quite relevant | | | Extremely relevant | | | Total | | | Yes/no (positive/negative) | |
|  |  |  |  | n | (%) | n | (%) | | n | (%) | | n | (%) | | N | (%) | |  |
| 1. Fall in older adults ≥ 65 | | | | 0 | (0) | 1 | (10) | | 5 | (50) | | 4 | (40) | | 10 | (100) | | Yes (positive) |
| 1. Location of injury | | | | 0 | (0) | 3 | (30) | | 5 | (50) | | 2 | (20) | | 10 | (100) | | Yes (positive) |
| 1. Reason for fall | | | | 0 | (0) | 1 | (10) | | 5 | (50) | | 4 | (40) | | 10 | (100) | | Yes (positive) |
| 1. Determining level of urgency and if for a low urgency case there is a possibility to dispatch GP for home visit (rather than immediate hospital visit) following fall in older adults | | | | 0 | (0) | 3 | (30) | | 3 | (30) | | 4 | (40) | | 10 | (100) | | Yes (positive) |
| Note:  *GP=* General Practitioner | | | | | | | | | | | | | | | | |  | |

|  | | | | | | | | | | | | | | | | |  | |
| --- | --- | --- | --- | --- | --- | --- | --- | --- | --- | --- | --- | --- | --- | --- | --- | --- | --- | --- |
| Protocol on “Cardiac arrest – deceased” | | | | | | | | | | | | | | | | |  | |
|  | | | | Relevance | | | | | | | | | | | | | Consensus status | |
| Item |  |  |  | Not relevant at all | | Somewhat relevant | | Quite relevant | | | Extremely relevant | | | Total | | | Yes/no (positive/negative) | |
|  |  |  |  | n | (%) | n | (%) | | n | (%) | | n | (%) | | N | (%) | |  |
| 1. Nature of death (patient in a palliative setting, expected vs unexpected death, do not resuscitate, etc) | | | | 0 | (0) | 2 | (20) | | 1 | (10) | | 7 | (70) | | 10 | (100) | | Yes (positive) |
| 1. Practical questions to better evaluate whether to dispatch MUG or GP (i.e. If the patient is in a palliative care setting or DNR code, should there be an option to send GP (rather than MUG)?) | | | | 0 | (0) | 0 | (0) | | 3 | (30) | | 7 | (70) | | 10 | (100) | | Yes (positive) |
| Note: *GP=* General Practitioner; *DNR=* Do Not Resuscitate*; MUG* = Mobile Emergency Group | | | | | | | | | | | | | | | | |  | |

|  | | | | | | | | | | | | | | | | |  | |
| --- | --- | --- | --- | --- | --- | --- | --- | --- | --- | --- | --- | --- | --- | --- | --- | --- | --- | --- |
| Protocol on “Urogenital problems” | | | | | | | | | | | | | | | | |  | |
|  | | | | Relevance | | | | | | | | | | | | | Consensus status | |
| Item |  |  |  | Not relevant at all | | Somewhat relevant | | Quite relevant | | | Extremely relevant | | | Total | | | Yes/no (positive/negative) | |
|  |  |  |  | n | (%) | n | (%) | | n | (%) | | n | (%) | | N | (%) | |  |
| 1. Problems with use of other medical devices in older adults ≥ 65 (i.e. use of urinary catheter, stomach catheter, etc). | | | | 0 | (0) | 4 | (40) | | 5 | (50) | | 1 | (10) | | 10 | (100) | | No |
| 1. Urinary incontinence (i.e. is it the first time, how long since start of problem, blood in urine) | | | | 0 | (0) | 3 | (30) | | 6 | (60) | | 1 | (10) | | 10 | (100) | | Yes (positive) |
| 1. Urinary overflow | | | | 1 | (10) | 2 | (20) | | 3 | (30) | | 4 | (40) | | 10 | (100) | | Yes (positive) |
| 1. Possibility to send patient a GP for home visit (rather than only immediate hospital visit) following urinary retention for 6hrs and more | | | | 2 | (20) | 1 | (10) | | 3 | (30) | | 4 | (40) | | 10 | (100) | | Yes (positive) |
| 1. Removal of questions related to temperature and urinary retention from this protocol and added to "confusion" protocol instead | | | | 6 | (60) | 1 | (10) | | 2 | (20) | | 1 | (10) | | 10 | (100) | | Yes (negative) |
| Note: *GP=* General Practitioner | | | | | | | | | | | | | | | | |  | |

|  | | | | | | | | | | | | | | | | |  | |
| --- | --- | --- | --- | --- | --- | --- | --- | --- | --- | --- | --- | --- | --- | --- | --- | --- | --- | --- |
| Protocol on “Cardiac problem other than thoracic pain” | | | | | | | | | | | | | | | | |  | |
|  | | | | Relevance | | | | | | | | | | | | | Consensus status | |
| Item |  |  |  | Not relevant at all | | Somewhat relevant | | Quite relevant | | | Extremely relevant | | | Total | | | Yes/no (positive/negative) | |
|  |  |  |  | n | (%) | n | (%) | | n | (%) | | n | (%) | | N | (%) | |  |
| 1. Swollen leg in older adults ≥ 65 | | | | 3 | (30) | 4 | (40) | | 1 | (10) | | 2 | (20) | | 10 | (100) | | Yes (negative) |
| 1. Shortness of breath in older adults ≥ 65 | | | | 2 | (20) | 3 | (30) | | 2 | (20) | | 3 | (30) | | 10 | (100) | | No |
| 1. Irregular heart palpitations in older adults ≥ 65 | | | | 2 | (20) | 6 | (60) | | 1 | (10) | | 1 | (10) | | 10 | (100) | | Yes (negative) |
| 1. Respiratory problems in older adults ≥ 65 | | | | 2 | (20) | 5 | (50) | | 1 | (10) | | 2 | (20) | | 10 | (100) | | Yes (negative) |
| 1. Possibility to push dimple into swollen leg (possibly indicating heart failure) | | | | 2 | (20) | 4 | (40) | | 3 | (30) | | 1 | (10) | | 10 | (100) | | No |
| 1. Possibility to push dimple into leg given shortness of breath | | | | 1 | (10) | 3 | (30) | | 6 | (60) | | 0 | (0) | | 10 | (100) | | No |
| 1. Orthostatic hypotension | | | | 1 | (10) | 6 | (60) | | 3 | (30) | | 0 | (0) | | 10 | (100) | | Yes (negative) |
| 1. Addition of swollen legs complaint to protocol for hot/cold limbs | | | | 1 | (11.1) | 3 | (33.3) | | 2 | (22.2) | | 3 | (33.3) | | 9 | (100) | | No |
| 1. Adapting current 1733 protocol for "syncope" to broader guidelines for “transit loss of consciousness” (as recently adapted by the European Society of Cardiology {Brignole, 2018 #2390} | | | | 2 | (22.2) | 4 | (44.4) | | 3 | (33.3) | | 0 | (0) | | 9 | (100) | | No |
|  | | | | | | | | | | | | | | | | |  | |

|  | | | | | | | | | | | | | | | | |  | |
| --- | --- | --- | --- | --- | --- | --- | --- | --- | --- | --- | --- | --- | --- | --- | --- | --- | --- | --- |
| Protocol on “Nose-throat-ear-tooth” | | | | | | | | | | | | | | | | |  | |
|  | | | | Relevance | | | | | | | | | | | | | Consensus status | |
| Item |  |  |  | Not relevant at all | | Somewhat relevant | | Quite relevant | | | Extremely relevant | | | Total | | | Yes/no (positive/negative) | |
|  |  |  |  | n | (%) | n | (%) | | n | (%) | | n | (%) | | N | (%) | |  |
| 1. Tooth problems for all ages (separate from nose-throat-ear protocol) | | | | 5 | (50) | 1 | (10) | | 3 | (30) | | 1 | (10) | | 10 | (100) | | No |
| 1. Location of pain | | | | 1 | (10) | 4 | (40) | | 3 | (30) | | 2 | (20) | | 10 | (100) | | No |
| 1. Problems related to swallowing foods | | | | 1 | (11.1) | 1 | (11.1) | | 5 | (55.6) | | 2 | (22.2) | | 9 | (100) | | Yes (positive) |
| 1. Clarification of GP's role for patients with tooth problems (whether there should be a possibility to dispatch patient to an on-call dentist) | | | | 0 | (0) | 1 | (11.1) | | 2 | (22.2) | | 6 | (66.7) | | 9 | (100) | | Yes (positive) |
| Note: *GP=* General Practitioner | | | | | | | | | | | | | | | | |  | |
|  | | | | | | | | | | | | | | | | |  | |
| Protocol on “Hot or cold limb” | | | | | | | | | | | | | | | | |  | |
|  | | | | Relevance | | | | | | | | | | | | | Consensus status | |
| Item |  |  |  | Not relevant at all | | Somewhat relevant | | Quite relevant | | | Extremely relevant | | | Total | | | Yes/no (positive/negative) | |
|  |  |  |  | n | (%) | n | (%) | | n | (%) | | n | (%) | | N | (%) | |  |
| 1. Swollen legs joints for all ages | | | | 2 | (20) | 3 | (30) | | 3 | (30) | | 2 | (20) | | 10 | (100) | | No |
| 1. Pain severity and changes in pain severity when lifting leg up and down (in the case of painful leg) | | | | 0 | (0) | 6 | (60) | | 2 | (20) | | 2 | (20) | | 10 | (100) | | No |
| 1. Integration of swollen legs or joints complaint into current protocol for hot or cold limb | | | | 1 | (10) | 4 | (40) | | 3 | (30) | | 2 | (20) | | 10 | (100) | | No |
|  | | | | | | | | | | | | | | | | |  | |

|  | | | | | | | | | | | | | | | | |  | |
| --- | --- | --- | --- | --- | --- | --- | --- | --- | --- | --- | --- | --- | --- | --- | --- | --- | --- | --- |
| Protocol on “Non-traumatic back pain” | | | | | | | | | | | | | | | | |  | |
|  | | | | Relevance | | | | | | | | | | | | | Consensus status | |
| Item |  |  |  | Not relevant at all | | Somewhat relevant | | Quite relevant | | | Extremely relevant | | | Total | | | Yes/no (positive/negative) | |
|  |  |  |  | n | (%) | n | (%) | | n | (%) | | n | (%) | | N | (%) | |  |
| 1. Location and severity of pain (to exclude potential diagnosis of a rupturing aneurysm) | | | | 1 | (10) | 3 | (30) | | 3 | (30) | | 3 | (30) | | 10 | (100) | | No |
| 1. Back problems | | | | 1 | (11.2) | 4 | (44.4) | | 4 | (44.4) | | 0 | (0) | | 9 | (100) | | No |
|  | | | | | | | | | | | | | | | | |  | |
